# Investigating the Clinico-Demographic Characteristics of Dengue Fever and Its Seroprevalence at a Tertiary Care Hospital in Northern India

**DOI:** 10.7759/cureus.57640

**Published:** 2024-04-05

**Authors:** Shefali Gupta, Akhalesh Shakya, Namita Mishra, Sana Islahi, Sweta Singh, Soumyabrata Nag, Arti Dwivedi, Mukesh Shukla, Somdatt Sen, Priyanka Dwivedi, Mayank Agarwal, Anirudh Mukherjee

**Affiliations:** 1 Microbiology, All India Institute of Medical Sciences, Raebareli, IND; 2 Paediatrics, All India Institute of Medical Sciences, Raebareli, IND; 3 Microbiology, All India Institute of Medical Sciences, Nagpur, IND; 4 Virus Research and Diagnostic Laboratory, All India Institute of Medical Sciences, Raebareli, IND; 5 Community and Family Medicine, All India Institute of Medical Sciences, Raebareli, IND; 6 Physiology, All India Institute of Medical Sciences, Raebareli, IND; 7 Internal Medicine, All India Institute of Medical Sciences, Raebareli, IND

**Keywords:** dengue serotype, real-time polymerase chain reaction (rt-pcr), igm elisa, dengue ns1, enzyme linked immunosorbent assay (elisa), dengue virus serotype

## Abstract

Background and objective

Dengue virus (DENV) is a major global health threat, causing over 50,000 deaths annually. The state of Uttar Pradesh (UP) in India faces significant challenges due to the increasing number of dengue cases detected. This study aimed to assess DENV seropositivity in the Raebareli district of UP, to offer crucial insights into the region's effective control and management strategies.

Materials and methods

This study, after obtaining approval from the ethics committee, analyzed blood samples of individuals suspected of having dengue at a teaching hospital in rural UP between January and December 2022. To determine the disease's seroprevalence, both dengue NS1 antigen ELISA and dengue IgM Microlisa were conducted. Furthermore, RT-PCR was performed on NS1-positive samples to confirm the serotypes. The collected data were analyzed using Epi Info 7.0.

Results

Of the 589 suspected dengue cases, 86 (14.60%) tested positive for dengue NS1 and/or IgM. Our findings showed that males (n=330, 56.03%) and adolescents and young adults (n=301, 51.1%) from rural areas (n=523, 88.4%) were predominantly affected. Cases peaked post-monsoon, and platelet levels were notably low in NS1-positive cases. Dengue serotype 2 (DEN-2) was found in all RT-PCR-positive samples. Our results revealed a dengue seroprevalence of 14.60% (n=86), which peaked in post-monsoon months. The higher incidence among males and young adults from rural areas attending the outpatient department highlights the importance of targeted interventions and community surveillance. RT-PCR confirmed the circulation of a single serotype in the region.

Conclusions

This study contributes crucial insights into dengue's epidemiology and clinical profile and its findings are all the more significant now as India prepares for phase 3 trials of a quadrivalent dengue-virus vaccine in 2024. Adolescent and young adult males have an increased likelihood of acquiring the virus, and this demographic can be prioritized for vaccine trials.

## Introduction

The dengue virus (DENV) infection is a mosquito-borne viral infection that poses a significant global health threat. It has a high prevalence rate, affecting over 50% of the world's population, and it is often associated with severe complications and fatalities. The incidence of dengue has dramatically increased in recent years, with cases reported to WHO surging from approximately 0.5 million in 2000 to 5.2 million in 2019 [1,2]. This serious viral disease is caused by any of the four serotypes of dengue virus: DEN-1 through DEN-4. It exhibits a broad range of clinical symptoms, varying from mild fever to severe plasma leakage, which may lead to life-threatening shock [3]. Within the past decade, Malaysia has reported the emergence of DEN-5, the fifth serotype of the dengue virus. Unlike the other four serotypes, DEN-5 follows a sylvatic cycle and exhibits a lower transmission rate [4].

DENV is a widespread infection in Southeast Asia and the Western Pacific, representing around 70% of the global disease burden. Most cases in Asia have been reported from Bangladesh (101,000), Malaysia (131,000), the Philippines (420,000), and Vietnam (320,000). India also continues to be a prominent location for dengue cases worldwide [2,5]. The epidemiology of dengue fever is undergoing significant developments, with more extensive and frequent outbreaks being recorded in urban and rural regions, attributable to each of the four serotypes in India [6]. Efforts are underway to improve dengue surveillance in India, but more seroepidemiological investigations are needed to estimate the disease burden accurately. However, in India, there has been limited research examining the prevalence of dengue, with a focus on metropolitan areas [7]. 

Currently, research on the prevalence of dengue fever in various demographic areas across India is scarce. An evaluation of the actual impact of dengue is imperative for the appropriate allocation of resources towards its monitoring and management. It is crucial to constantly monitor the transmission of DENV to implement an effective management strategy for the same [1]. Given the threat it poses to the country and the introduction of an indigenous dengue vaccine on the horizon, conducting seroprevalence studies becomes immensely important. Uttar Pradesh (UP), the most populated state in India, faces a substantial healthcare concern stemming from the dissemination of DENV, which is aggravated by inadequate sanitation and the high population density in the state. Moreover, it is worth noting that DENV is capable of triggering acute encephalitis syndrome (AES) [8]. 

In this study, we endeavor to ascertain the seropositivity of DENV infection among suspected cases visiting a teaching hospital in the Raebareli district, UP, north India. We believe our findings will provide fundamental insights into the types of DENV circulating in this region, thereby aiding in the development of effective control and management strategies against this vector-borne disease.

## Materials and methods

Study design, setting, and population

This study, duly approved by the Institutional Ethics Committee (IEC No.: 2023-13-OTH-EXP- 5), was a retrospective analysis carried out at the Viral Research and Diagnostic Laboratory (DHR/ICMR) in the Department of Microbiology at a teaching hospital in rural UP, India, from January 2022 to December 2022. Since the study was retrospective in nature and data were deidentified and stored as locked digital files after obtaining it, the Institutional Ethics Committee granted a waiver of consent. The study included patients of General Medicine and Paediatrics, either visiting the outpatient department (OPD) or admitted to the inpatient department (IPD) of AIIMS Raebareli, exhibiting clinical symptoms suggestive of dengue fever. Dengue was clinically suspected when a patient had a high fever (40 °C/104 °F) along with any two of the following symptoms during the febrile phase (two to seven days): severe headache, pain behind the eyes, muscle and joint pains, nausea, vomiting, swollen glands, and rash [2]. The exclusion criteria were as follows: individuals diagnosed with febrile illnesses such as malaria, typhoid, scrub typhus, chikungunya, leptospirosis, and any patient with a history of fever exceeding seven days.

Serum samples were collected and transported to the microbiology laboratory in a proper cold chain from clinically suspected dengue cases, as recommended by treating doctors; they were then analyzed for serological testing in the study. Patients' demographic data and laboratory parameters were collected from the hospital records. The serum samples positive for dengue NS1 and/or IgM were labeled laboratory-confirmed dengue cases.

Sample size

Assuming a dengue seroprevalence of 25%, a minimum sample size of 289 was considered with a 95% confidence interval and a 5% margin of error.

Serology laboratory processing

The serum samples were examined following standard laboratory procedures by subjecting them to both DEN NS1 antigen ELISA and a DEN IgM Microlisa kit (J. Mitra & Co. Pvt. Ltd., Bengaluru, India) developed to detect dengue virus (serotypes 1-4) according to the manufacturer's instructions. The serum samples were kept at -20 °C for further molecular studies.

Molecular studies

RT-PCR was performed on the NS1-positive samples. The viral RNA was extracted from dengue NS1-positive serum samples by using a TRUPCR viral nucleic acid extraction kit (3B BlackBio Biotech India Ltd., Bhopal, India) as per the kit protocol. The extracted RNA was stored at −80 °C until further use. The TRUPCR dengue virus serotyping kit (3B Blackbio Biotech India Ltd.) was used for dengue virus serotype confirmation. A multiplex RT-PCR assay was performed to detect the four serotypes as per the kit’s instructions. The RT-PCR amplification was carried out in two tubes for each sample; the first tube contained the enzyme master mix, primers for dengue virus, internal control, and dengue virus serotype 1, while the second tube contained the enzyme master mix and primers for dengue virus serotypes 2, 3, and 4. The instructions for the commercial RT-PCR kit for the serotyping of dengue virus were followed. The total volume of the reaction was 25 µl, which contained 10 µl of master mix, 4.65 µl of dengue primer probe mix, 0.35 µl of enzyme mix, and 10 µl of extracted RNA. Sterilized water (provided in the kit) was used as a negative control. RT-PCR was done in a CFX96 real-time system (Bio-Rad). The positive control was also provided in the kit. The thermal cycling conditions were as follows: hold at 20 min at 50 °C, 10 min at 94 °C, holding cycle 2: 15 sec at 94 °C; fluorescence acquisition at 45 sec at 55 °C and 15 sec at 72.0 °C for 45 cycles. The amplified products accounted for increased fluorescence detection in a specific channel.

Data analysis

Data were compiled and analyzed using the statistical software Epi Info version 7.0. Descriptive statistics such as frequency and percentage for categorical variables were determined. All quantitative values were expressed as mean ± standard deviation (SD). The association between sociodemographic variables and laboratory-confirmed and laboratory negatives among clinically suspected cases was assessed using the Chi-square test. Analysis of variance (ANOVA) was used to compare the mean values of platelets and WBC among different categories of serological results. A p-value <0.05 was considered statistically significant.

## Results

**Figure 1:**
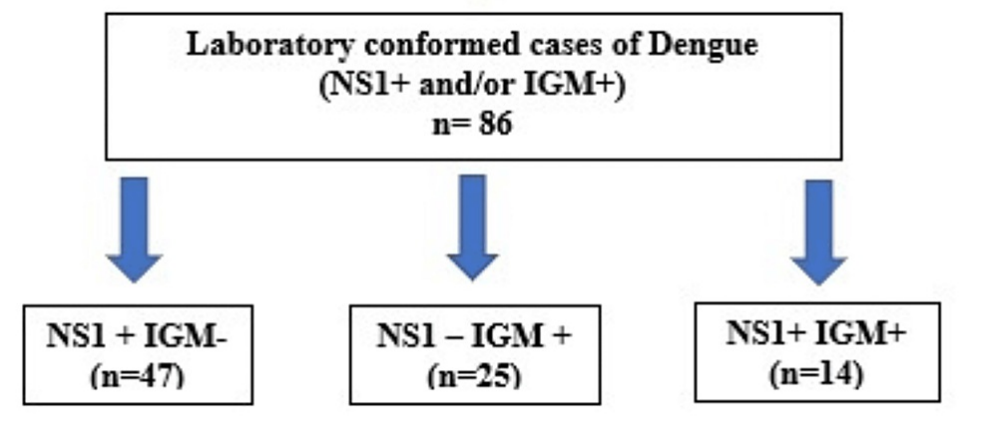
Distribution of dengue seropositive results NS1: dengue NS1; IgM: dengue IgM

A total of 882 patients with acute febrile illness presented to the Medicine and Paediatric Departments’ OPD or IPD during the study period. Of them, 293 were excluded based on their history, symptoms, and clinical examination. A total of 589 blood samples of suspected dengue cases were collected, of which 86 (14.60%) returned positive for dengue NS1 and/or IgM (Figure 1).

Most of the patients were male (n=330, 56.03%); adolescents and young adults accounted for over half of the cohort (n=301, 51.1%); 85.39% (n=503) of the samples were obtained from OPD, while 88.79% (n=523) of the patients were from rural areas. A higher seroprevalence was closely linked to the above-mentioned demographic parameters. The distribution of patients and dengue-positive cases by demographic characteristics is presented in Table 1.

**Table 1:** Demographic distribution of the study population NS1: dengue NS1; IgM: dengue IgM; OPD: outpatient department; IPD: inpatient department

Variable	Laboratory-confirmed cases (NS1+ and/or IGM+) (n=86), n (%)	Laboratory negatives among probable cases (n=503), n (%)	*p*-value
Age group, years			
0-10	6 (7.0%)	96 (19.1%)	0.08
11-20	27 (31.4%)	118 (23.5%)	
21-30	25 (29.1%)	131 (26.0%)	
31-40	13 (15.1%)	55 (10.9%)	
41-50	6 (7.0%)	42 (8.3%)	
>50	9 (10.5%)	61 (12.1%)	
Gender			
Male	59 (68.6%)	271 (53.9%)	0.007
Female	27 (31.4%)	232 (46.1%)	
Residence			
Urban	10 (11.6%)	56 (11.1%)	0.50
Rural	76 (88.4%)	447 (88.9%)	
Patient source			
OPD	76 (88.4%)	427 (84.9%)	0.25
IPD	10 (11.6%)	76 (15.1%)	

**Figure 2:**
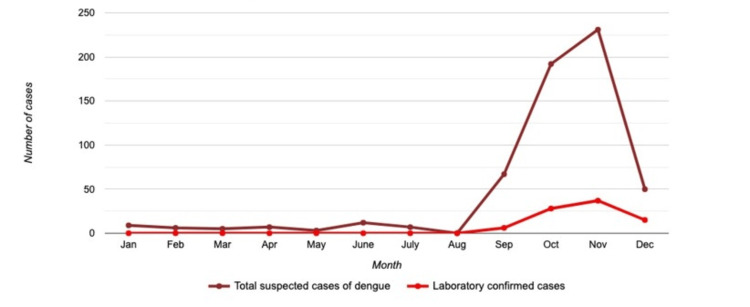
Month-wise distribution of dengue cases

The number of suspected and laboratory-confirmed dengue cases began to rise in September; it went on to peak in November and decline again in December. This coincides with the post-rainy season in the region where the study was conducted (Figure 2).

No consistent symptom was found in all dengue-positive cases except fever. Various symptoms such as headaches, rashes, and joint pain were each present in about 40-55% of the cases, as shown in Table 2.

**Table 2:** Distribution of symptoms among dengue-positive cases

Symptoms	N (%), (n=86)
Headache	46 (53.49%)
Retro-orbital pain	35 (40.69%)
Muscle and joint pains	48 (55.81%)
Nausea, vomiting	38 (44.18%)
Swollen glands	41 (47.67%)
Rash	38 (44.18%)

It was observed that platelet count and WBC levels were significantly low among dengue-positive cases, especially if the patient was positive for NS1 with or without IgM positivity (Table 3).

**Table 3:** Platelet count and WBC distribution in dengue seropositive results *p*-value for platelet count: 0.04; *p*-value for WBC: 0.03 NS1: dengue NS1; IgM: dengue IgM; SD: standard deviation; WBC: white blood cells

Serological results	Platelet counts (lakhs), mean ± SD	WBC (thousands), mean ± SD
NS1+ IGM+ (n=14)	2.01 ± 0.94	11.00 ± 5.54
NS1+ IGM- (n=47)	2.63 ± 1.24	11.32 ± 2.52
NS1- IGM+ (n=25)	3.10 ± 1.09	12.53 ± 3.29
NS1- IGM- (n=503)	3.37 ± 7.07	12.83 ± 3.13

All the NS1-positive samples were subjected to RT-PCR to determine the serotype of the virus (Table 4). All 32 (52.46%) RT-PCR-positive samples belonged to dengue serotype 2.

**Table 4:** RT-PCR findings among NS1-positive samples RT-PCR: real-time polymerase chain reaction; NS1: dengue NS1

RT-PCR results	NS1-positive, n (%)
RT-PCR-positive	32 (52.46%)
RT-PCR-negative	29 (47.54%)
Total	61 (100%)

## Discussion

This study explores dengue seroprevalence and epidemiology in the Raebareli district and its surrounding areas in central UP, north India. The demographic distribution of the patients sheds light on the potential risk factors and provides insights into structuring public health strategies. Our study found that in 2022, 14.60% (86/589 cases) were positive for dengue. In the post-monsoon months of September to December, dengue fever cases spiked due to the reduced extrinsic incubation time of DENV, which is attributable to meteorological variables, including ambient temperatures and rainfall. Similar peaks were observed in various studies across India [9-11]. Research shows that Delhi and Rajasthan also experience a peak season for dengue during the same period [12].

A notable finding involves the significantly higher prevalence of dengue-positive cases in males compared to females (68.6% vs. 31.4%). Several studies have shown that males are more susceptible to dengue fever than females, including those conducted in Malaysia, western Uttar Pradesh, and other parts of India [12-14]. We must acknowledge that cultural and societal factors might have led to this imbalance, and the fact that men spend more time engaged in outdoor activities could have also played a role. A study by Hsu et al. from Taiwan did not find any difference in the seroprevalence of dengue between the genders, while Alagarasu et al. reported a higher seroprevalence among females in their study [1,15].

Regarding the age distribution in our study, adolescents and young adults comprised more than half of the cases. Specifically, the age groups 11-20 years and 21-30 years accounted for a significantly higher number of laboratory-confirmed dengue cases. Dengue fever is most prevalent among people aged 11-30 years, according to research conducted in various regions of India, including Odisha, Delhi, West Bengal, and other areas of UP [16-19]. The higher trends of positive dengue cases among males and young adults may be linked to social, occupational, and behavioral factors, such as increased outdoor activities and exposure to mosquito vectors. The underlying reasons for this gender disparity and its implications for targeted intervention strategies should be further investigated. Public health campaigns and preventive measures should consider targeting these demographic groups to effectively curb the spread of dengue.

Our findings contrast with the higher prevalence of the condition in adults and the elderly reported by Suresh et al. and Hsu et al., which could be attributed to the specific demographic characteristics of the study population [14,15,19]. Most samples (85.39%) were collected from OPD, indicating that a substantial proportion of dengue cases were mild and managed in outpatient settings; thus, the importance of increasing dengue surveillance in the community must be emphasized. Additionally, the preponderance of cases in rural areas underscores the need for targeted interventions in these regions. Specific environmental and socioeconomic factors contributing to dengue transmission in rural areas must be addressed promptly to prevent an outbreak.

Notably, no statistically significant differences were observed between patients in urban and rural areas or between those who sought outpatient care and those who were admitted as inpatients. This suggests that the risk of contracting dengue is not solely contingent upon the patient's place of residence or the healthcare setting, indicating the wide prevalence of the disease. Approximately 11% of positive cases were from inpatient admissions, signifying a heightened risk of hospitalization due to dengue. Additional research is needed to examine the prospective variables that impact the magnitude of the ailment.

Dengue fever typically presents with fever as its primary symptom, making it challenging to diagnose it without laboratory tests. This is especially important in areas where other diseases that cause fever, such as malaria, Japanese encephalitis, chikungunya, and enteric fever, are prevalent. Healthcare providers may offer supportive care for dealing with various clinical symptoms of dengue infection by recognizing the vast array of symptoms attributed to the virus. Thrombocytopenia frequently occurs in patients with dengue fever, and this condition may offer significant clues to diagnosing and treating it. The relationship between NS1 positivity and low platelet count further bolsters this link, underlining the importance of NS1 antigen testing in early diagnosis of dengue fever. Kulkarni et al. have also made similar observations in their research [20].

When patients still have a high fever during the early stages of the infection, DENV RNA and NS1 protein are detected; however, DENV RNA disappears from the bloodstream before NS1 protein. The late convalescent sera exhibit improved sensitivity to NS1 [21]. Thus, we performed RT-PCR on NS1-positive samples only. We identified 52.46% (N=32) of the NS1-positive samples as DEN-2, with the remaining samples testing negative by RT-PCR. This suggests that only one dengue virus serotype is circulating among the tested population. DEN-2 was the most common strain of DENV in circulation in UP during 2020 and 2021 [12]. These findings align with those by Deval et al. in their study from eastern UP and Bihar [6]. DEN-2 exhibits enhanced adaptations for transmission by mosquitoes [22]. Between 1990 and 2015, research indicated that DEN-2 significantly contributed to numerous global dengue virus infections [23].

On the other hand, Behera et al. showed the presence of all four dengue serotypes among their study population from eastern UP [24]. Different serotypes and genotypes of dengue virus may exhibit variations in clinical severity and immune response [25]. Accurate serotype identification thus aids in tailoring appropriate medical treatments and vaccine development efforts, which is significant in the context of India beginning phase 3 trials for a quadrivalent dengue-virus vaccine in 2024 [26].

## Conclusions

Our research elucidated the epidemiological and clinical dimensions of dengue fever outbreaks documented in the Raebareli district of UP in 2022. Our study found that the DEN-2 was responsible for the outbreaks during the monsoon season. Our findings also showed that DENV was no longer confined to urban areas; it was proliferating in the rural regions of UP in northern India, with a noteworthy incidence of attacks. Males demonstrate a greater vulnerability to infection in comparison to females, along with those in the age group of 11-30 years. Hence, this demographic can be prioritized for vaccine trials.
